# Emergency Department Visits Related to Cirrhosis: A Retrospective Study of the Nationwide Emergency Department Sample 2006 to 2011

**DOI:** 10.1097/MD.0000000000000308

**Published:** 2015-01-09

**Authors:** Chaitanya Pant, Mojtaba Olyaee, Richard Gilroy, Prashant K. Pandya, Jody C. Olson, Melissa Oropeza-Vail, Tarun Rai, Abhishek Deshpande

**Affiliations:** From the Division of Gastroenterology, Hepatology and Motility, Department of Internal Medicine, University of Kansas Medical Center, Kansas City, KS (CP, MO, RG, PKP, JCO, MOV, TR); and Medicine Institute Center for Value Based Care Research, Cleveland Clinic, Cleveland, OH (AD).

## Abstract

There is scant literature about cirrhosis and its associated complications in a non-hospitalized population.

To study the epidemiology of cirrhosis-associated Emergency Department visits in the US.

Estimates were calculated in patients’ ≥18 years using the Nationwide Emergency Department Sample.

The number of visits associated with an International Classification of Diseases-9 diagnosis code of cirrhosis increased non-significantly from 23.81/10,000 population (2006) to 23.9/10,000 population (2011; *P* = 0.05). A majority of these patients (75.30%) underwent hospital admission, the greatest risk factor for this was the presence of ≥3 comorbidities (adjusted odds ratio 30.8; 95% Confidence Interval 30.4–31.2). Infection was the most frequent concurrent complicating diagnosis associated with cirrhosis (20.1%). There was a decreased incidence in most of the complicating conditions except for hepatorenal syndrome and spontaneous bacterial peritonitis.

Our results indicate a stable trend for cirrhosis-associated Emergency Department visits from 2006 to 2011. Further studies are required to investigate the increased incidence of spontaneous bacterial peritonitis and hepatorenal renal syndrome in the cirrhotic population.

## INTRODUCTION

Cirrhosis is the eighth leading cause of death in the US and in 2010 accounted for approximately 49,500 deaths, increased from 35,500 deaths in 1990.^1^ Patients with cirrhosis carry an increased burden of comorbidities, like depression, drug addiction and coronary artery disease and have cirrhosis-related complications including ascites, hepatorenal syndrome (HRS), esophageal variceal bleeding (EVB), and hepatic encephalopathy (HE).^2^ Recent analyses of a national US database indicate a substantial and increasing prevalence of cirrhosis-related complications in hospitalized inpatients.^3–5^

However, these results relate to a cohort of cirrhotic patients undergoing inpatient treatment and are not necessarily representative of the disease as it occurs in the outpatient setting. We therefore interrogated a national US Emergency Department (ED) database over a 5-year period from 2006 to 2011 to gain further insight into the epidemiology of cirrhosis and its associated complications in a non-hospitalized population.

## MATERIALS AND METHODS

### Data Source

We used the Healthcare Cost and Utilization Project Nationwide Emergency Department Sample (HCUP-NEDS) years 2006 to 2011 for our study. The 2011 HCUP-NEDS comprises approximately 29.5 million ED visits sampled from 951 hospitals in 30 US states representing a 20% stratified sample of US hospital-based EDs. Weights are provided to calculate national estimates pertaining to approximately 131 million ED visits in 2011. The NEDS contains event-level records, not patient-level records; individual patients who visit the ED more than once in a year may be recorded in the NEDS multiple times. Each ED visit entry contains 1 primary discharge diagnosis, 1 to 14 secondary diagnoses (based on the International Classification of Diseases, Ninth Revision, Clinical Modification [ICD-9-CM] diagnosis codes), demographic information, and details of disposition from the ED. Ethical approval was therefore not necessary for the conduct of this study.

### Variable Definition

Adult patients were identified starting at age 18 years. We extracted all entries with a primary or secondary discharge diagnosis of cirrhosis (ICD-9-CM codes: 5712, 5715, 5716). Age, sex, insurance status, household income, geographic location of care, and hospital setting were also obtained for the extracted cases. Cases were then queried for complications that are known to occur frequently in patients with cirrhosis. These included infections; urinary tract infection (UTI), skin and subcutaneous tissue infections (SSCI), spontaneous bacterial peritonitis (SBP), *Clostridium difficile* infection (CDI), or pneumonia were identified using discharge ICD-9-CM codes: UTI (1122, 59010–11, 5902–03, 59080–81, 5950, 5970, 5990); SSCI (680–82, 684, 686); SBP (56723, 5672); CDI (00845), and pneumonia (480–83, 487). Other complications also queried for included HE, HRS, and EVB, which were identified using discharge ICD-9-CM codes: HE (5722), HRS (5724), and EVB (4560, 45620).

To assess independent risk factors associated with hospital admission, we assessed and controlled for the presence of comorbidities in our study population. The examined comorbid conditions consisted of 28 disease states contained within the Elixhauser comorbidity index minus the presence of liver disorders.^6^ This is a widely used index in which higher scores indicate a greater comorbid disease burden.^7,8^

### Statistical Analysis

Statistical analyses were performed using SAS version 9.3 (SAS Institute, Cary, NC). The rate of cirrhosis-associated ED visits was expressed per 10,000 census population, thereby adjusting for the increase in the US population over time. Data were obtained from the US Census Bureau, Population Division, Annual Estimates of the Population for the US, Regions, and Divisions and US Census Bureau, Current Population Reports. The Chi-square test was used to compare categorical variables and the 2-proportion *Z*-test was used to compare rates. Univariate and multiple variable logistic regression models were used to study the association of selected variables of interest with the outcome of admission to the hospital from the ED. For trend analysis, we used the Cochran–Armitage test. The threshold for significance for all analyses was *P* < 0.01.

## RESULTS

During the calendar years 2006 to 2011, there were an estimated total of 3,127,986 ED visits associated with an ICD-9-CM diagnosis code of cirrhosis. Table 1 details the demographic- and hospital-related characteristics of this patient population. The median age of patients was 56 years (interquartile range (IQR) 16) and 61.8% were male compared to 38.20% female patients (*P* < 0.01). Socioeconomic analyses revealed that 65.20% patients had either Medicare or Medicaid insurance while 31.80% belonged to the lowest national income quartile. The southern region of US accounted for 37.20% of all cirrhosis-associated ED visits. Visits to non-teaching hospitals were more frequent than teaching hospitals (55.70% vs 44.30%; *P* < 0.01) and most facilities were located in urban areas (84.40%; *P* < 0.01).

**TABLE 1:**
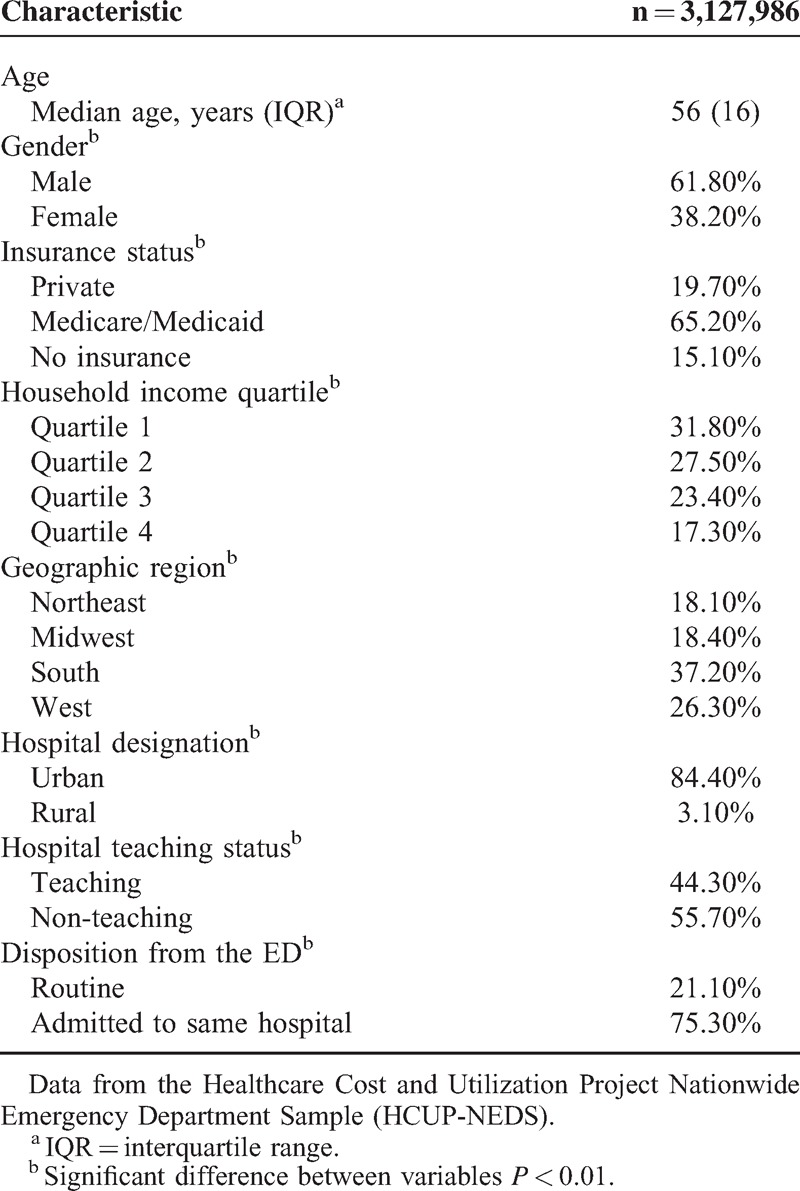
Demographic- and Hospital-Related Characteristics of Patients Ages 18 Years and Above With a Primary or Secondary Discharge Diagnosis of Cirrhosis Who Visited an Emergency Department (ED) in the US From 2006 to 2011

Regarding the disposition of the patients from the ED, the majority were admitted to the same hospital with a smaller number of routine discharges (75.30% vs 21.10%; *P* < 0.01). Using multiple variable logistic regression we identified factors that were associated with an increased rate of hospital admission for cirrhosis (Table 2). The greatest risk factors were the presence of ≥3 comorbid conditions (adjusted odds ratio [aOR] 30.8; 95% CI 30.4–31.2), the presence of cirrhosis-associated complications: EVB (aOR 13.9; 95% CI 13.5–14.3), HRS (aOR 5.6; 95% CI 5.4–5.8), HE (aOR 4.4; 95% CI 4.3–4.5), *any* infection (aOR 3.8; 95% CI 3.7–3.8), and presentation to an ED in the north-east region of the US (aOR 2.3; 95% CI 2.2–2.3).

**TABLE 2:**
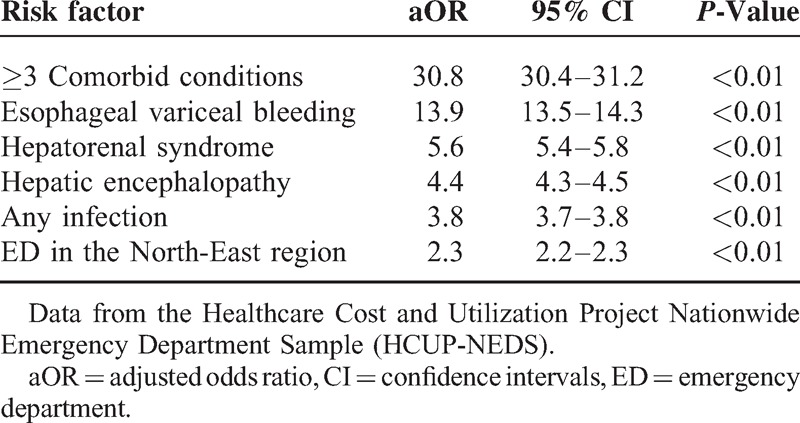
Risk Factors Associated With Hospital Admission in Patient With Cirrhosis-Associated Emergency Department (ED) Visits Using a Multiple Variable Regression Model in Patients Ages 18 Years and Above From 2006 to 2011

An analysis of temporal trends during the period of our study revealed that cirrhosis-associated ED visits increased slightly from 23.81/10,000 population in 2006 to 23.9/10,000 population in 2011; a non-significant increase of 0.4% (*P* = 0.05). In a similar period of time for a corresponding age group, the rate of *all* ED visits increased from 4167.3/10,000 population in 2006 to 4382.5/1000 population in 2011. This represented an increase of 5.2% and indicated an overall increased trend for all ED visits from 2006 to 2011 (*P* < 0.01).

We next determined the co-occurrence of cirrhosis-related complications in our patient population (Table 3). The most frequent category of complicating diagnoses was the presence of any infection (20.1%); UTI was the most common specific infection with an incidence of 10.9%. HE was the next most commonly occurring complication (15.5%) followed by EVB (6.0%) and HRS (4.0%). In the period from 2006 to 2011, there was a decrease in the incidence of both overall and individual complicating infections (*P* < 0.01) except for SBP, which increased by 14.7% (*P* < 0.01). Concurrently, there was also a significant decrease in the rates of EVB (23.2%; *P* < 0.01) and HE (17.7%; *P* < 0.01), although the rate of HRS did increase by 25.7% (*P* < 0.01).

**TABLE 3:**
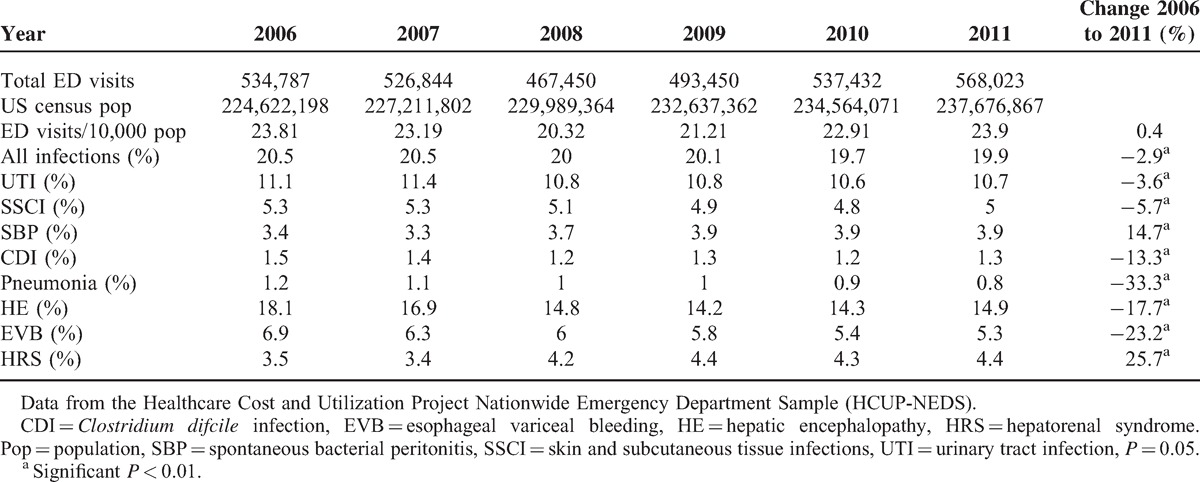
Rates of Cirrhosis-Associated Emergency Department (ED) Visits and Complicating Diagnoses in Patients Ages 18 Years and Above From 2006 to 2011

## DISCUSSION

Our study of the NEDS database demonstrates that the population-adjusted rate of cirrhosis-associated ED visits remained largely stable from 2006 to 2011. Contemporaneously, the population-adjusted rate of *all* ED visits increased by 5% with an overall increased trend. An examination of the demographic characteristics of the study population highlights a few noteworthy features. First, 37.2% of cirrhosis-associated ED visits were to hospitals in the southern region of the US. Second, 31.8% of the study population belonged to the lowest national income quartile. Last, approximately three-fourths of these patients were admitted to the same hospital, in contrast the admission rate for *all* ED visits ages 18 and above was approximately 15%. The disparate rate of cirrhosis in the lowest economic strata of society is an area of concern and merits further research relating to potential barriers in accessing treatment by these patients.^4^

Infection was overall the most frequent diagnostic category (20.1%) of cirrhosis-associated complications. Patients with cirrhosis are considered to suffer an acquired immunocompromised state and are particularly susceptible to infections, which account for higher mortality, increased renal insufficiency and greater utilization of healthcare resources.^3,9–11^ Our results indicate that from 2006 to 2011, the rates of pneumonia, UTI, SSCI, and CDI all decreased significantly; this was particularly evident in the case of pneumonia, which decreased by 33.3%. Interestingly, a recent analysis of the NEDS observed that the national rate of *all* ED visits due to pneumonia remained stable from 2006 to 2009.^12^

There was also a contemporaneous decrease in the rates of HE (17.7%) and EVB (23.2%) during the period of our study. While we can only speculate regarding the causes resulting in these decreased rates; the proliferating role of endoscopic variceal ligation and rifaximin as frontline therapies may have contributed in some measure to this decline. More concerning, is a significant increase in the rate of SBP (14.7%) and HRS (25.7%) in our study population. Unfortunately, the limitations of NEDS preclude further investigation regarding the basis for this escalation.

There are several limitations to our study. First, the NEDS contains encounter-level data, therefore, we are unable to assess for hospital readmissions or make patient-level associations across discharges and 1 patient may potentially account for several ED visits. Second, we have relied exclusively on ICD-9-CM codes for case identification. Third, the NEDS has limited clinical and demographic data, which limits our ability to investigate etiology. Fourth, there is a non-availability of data pertaining to Model For End-Stage Liver Disease (MELD) score calculations, medication, and antibiotic usage. Finally, our results represent a weighted estimate of national data.

Our analysis of the NEDS indicate a stable incidence of cirrhosis-associated ED visits in the period from 2006 to 2011 with a decreased rate of cirrhosis-related complications except for SBP and HRS. These data represent important complementary information to the study of the overall impact of cirrhosis in the US. Further research should be directed to investigating the increased trends observed for SBP and HRS in the cirrhotic population.
